# Cell–Cell Interaction of Macrophages and Vascular Smooth Muscle Cells in the Synthesis of Leukotriene B_4_

**DOI:** 10.1155/S0962935194000414

**Published:** 1994

**Authors:** M. Zou, C. Anges

**Affiliations:** 1 Laboratoire d'lmmunopharmacologie Institut des Cordeliers 15 rue de I'école de Mddecine Paris 75270 France; 2 Laboratoire de Biochimie Hôpital Broussais Paris 75674 France

## Abstract

Biosynthesis of LTB_4_ during cell-cell interaction between vascular smooth muscle cells (SMC) and alveolar macrophages (AM) has been investigated by use of both high-pressure Hquid chromatography (HPLC) and radtoimmunoassay (RIA). Both interleukin-β (IL-β) and tumour necrosis factor-α (TNFα) induced a time- and dose-dependent synthesis of 15-, and 5-hydroxyeicosatetraenoic acids (HETEs) from cultured SMC. However, neither TNFα nor IL-1β induced a significant LTB_4_ production in SMC alone or AM alone after 24 h of incubation. Addition of IL-1β and TNFα simultaneously to SMC resulted in a dose-dependent synergistic increase of HETEs. Macrophages dose-dependently transformed extremely low concentrations of exogenous LTA_4_ into LTB_4_. Incubation of vascular SMC with various numbers of AM in the presence of IL-1β (5 units/ml) and TNFα (10 units/ml) induced a great increase of LTB_4_ synthesis in comparison with the detectable levels of LTB_4_ produced by macrophages alone. Pretreatment of SMC with NDGA, cycloheximide, and actinomycin not only inhibited IL-1 and TNT induced HETEs synthesis but also abolished LTB_4_ production when co-incubated with macrophages. These results suggest that LTB_4_ in a mixture of SMC and macrophages could originate from a transcellular metabolism, i.e. macrophages transforming SMC-derived LTA_4_ into LTB_4_.


Mediators of Inflammation 3, 297–302 (1994)

### Cell–cell interaction of macrophages and vascular smooth muscle cells in the synthesis of leukotriene $B_4$

M. Zou[1,CA] and C. Anges[2]

[1]Laboratoire d'Immunopharmacologie, Institut des Cordeliers, 15 rue de l'école de Médecine 75270, Paris, France; [2]Laboratoire de Biochimie, Hôpital Broussais, 75674 Paris, France

[CA]Corresponding Author

**Biosynthesis of $LTB_4$ during cell–cell interaction between vascular smooth muscle cells (SMC) and alveolar macrophages (AM) has been investigated by use of both high-pressure liquid chromatography (HPLC) and radioimmunoassay (RIA). Both interleukin-1β (IL-1β) and tumour necrosis factor-α (TNFα) induced a time- and dose-dependent synthesis of 15-, and 5-hydroxy-eicosatetraenoic acids (HETEs) from cultured SMC. However, neither TNFα nor IL-1β induced a significant $LTB_4$ production in SMC alone or AM alone after 24 h of incubation. Addition of IL-1β and TNFα simultaneously to SMC resulted in a dose-dependent synergistic increase of HETEs. Macrophages dose-dependently transformed extremely low concentrations of exogenous $LTA_4$ into $LTB_4$. Incubation of vascular SMC with various numbers of AM in the presence of IL-1β (5 units/ml) and TNFα (10 units/ml) induced a great increase of $LTB_4$ synthesis in comparison with the detectable levels of $LTB_4$ produced by macrophages alone. Pretreatment of SMC with NDGA, cycloheximide, and actinomycin not only inhibited IL-1 and TNF induced HETEs synthesis but also abolished $LTB_4$ production when co-incubated with macrophages. These results suggest that $LTB_4$ in a mixture of SMC and macrophages could originate from a transcellular metabolism, i.e. macrophages transforming SMC-derived $LTA_4$ into $LTB_4$.**

**Key words:** Cell–cell interaction, Interleukin-1, Leukotriene, Macrophage, Smooth muscle cell, Tumour necrosis factor

### Introduction

The generation of leukotrienes (LTs) by lipoxygenase catalysed reactions is associated with a wide range of cell types that are involved in both physiological and pathological events.[1] The transcellular metabolism of lipoxygenase derived metabolites has recently been reported not only to amplify the level of eicosanoids within a local milieu but also to stimulate the generation of biologically active metabolites with functions different from those in its original cells. For example, the presence of erythrocytes can greatly induce $LTB_4$ formation from neutrophil-derived $LTA_4$;[2] co-incubation of endothelial cells,[3] smooth muscle cells[4] or platelets[3–8] with neutrophils, can result in transcellular $LTC_4$ synthesis. Perfusion of blood free rabbit lung and isolated pulmonary artery with neutrophils induced an overall $LTB_4$, $LTC_4$ and $LTD_4$ release.[9] The transfer of unstable metabolic intermediates has been considered to be a biochemical basis for these phenomena.[3–5,9,10]

Macrophages are well known to play a central role in the modulation of inflammatory and immune processes, as well as in tissue injury and repair.[11] Macrophages have 5-lipoxygenase activity that inserts molecular oxygen into arachidonic acid to form an unstable peroxide 5-HPETE leading to the generation of 5-HETE, or through the epoxide intermediate, $LTA_4$, to the generation of $LTB_4$ by hydrolase; or conjugation with glutathione by glutathione-*S*-transferase to form the cysteine $LTC_4$, $D_4$ and $E_4$.[12,13] Recently, many studies have indicated that macrophages are a potent source of arachidonate metabolites generated via lipoxygenase pathways.[13–15] *In vivo*, SMC and macrophages have been noted to interact at the site of thrombosis, vessel injury and inflammation by secreting interleukin-1 (IL-1), tumour necrosis factor (TNF), prostaglandins (PG) and leukotrienes.[15,16] This investigation was designed to study whether or not a shift in the metabolic profile of arachidonate products generated by activated macrophages or vascular SMC could be induced under circumstances that may be related more closely to those operative inflammatory reactions *in vivo*.

### Materials and Methods

*Reagents:* Human recombinant IL-1β and TNFα were obtained from Sigma (Saint Quentin Fallavier, France). Labelled $^3$H-AA and radioactive standards were from Amersham (Aylesbury, UK). Synthetic

© 1994 Rapid Communications of Oxford Ltd

$LTA_4$ methyl ester from Sigma was hydrolysed to yield the free acid according to the methods described by Maycock.[17] Octadecyl silica Sep-Pak cartridges were obtained from Millipore/Waters (Les Ulis, France). Media, sera and reagents for cell cultures were obtained, if not further specified, from Gibco (Paisley, UK). All solvents used in chromatographic systems were of HPLC grade.

*Vascular SMC culture:* Vascular SMC cells were obtained by dissociation of tissues from rat abdominal aorta with 0.05% EDTA and 0.1% trypsin in HAM F10 medium. During the first 2 weeks, the cells were cultured at 37°C, with 5% $CO_2$ in HAM F10 medium supplemented with 20% foetal calf serum. When a monolayer was obtained, the cells were removed by trypsin–EDTA (0.05–0.02%) and cultured in 25 ml flasks in HAM F10 medium, supplemented with 10% foetal calf serum and 1% penicillin–streptomycin. Culture SMC grew in typed 'hill and valley' formation. Cells up to the 25th passage were used for the experiments.

*Alveolar macrophage isolation and culture:* Respiratory disease-free 125 to 150 g female Wistar rats were housed under pathogen-free condition. Rats were anaesthetized with i.p. sodium pentobarbital and lungs were exercised and washed as described previously.[18] Bronchoalveolar lavage cells were 96% AM by microscopic examination of cytocentrifuge preparations stained with a modified Wright–Giemsa stain (Diff-Qick, American Scientific Products, IL). Bronchoalveolar cells ($5 \times 10^6$) suspended in 10 ml of M199 were plated in 25 ml flasks and cultured at 37°C in a humidified atmosphere of 5% $CO_2$ in air. After 2 h, nonadherent cells were removed by washing twice with the medium. Cell monolayers were then cultured in M199 containing 10% heat inactivated newborn calf serum before experimental incubation.

*$^3$H-arachidonic acid metabolism in macrophages and SMC:* Monolayer vascular SMC cells ($5 \times 10^6$) or macrophages ($5 \times 10^6$) were plated in a fresh culture medium. The cells were incubated with 1 μM $^3$H-AA for 18 h and then the medium was harvested. The cells were washed three times with PBS containing 0.25% fatty acid free bovine serum albumin (BSA) to eliminate non-incorporated AA. These cells were covered with 7 ml of serum free medium. IL-1β (5 units/ml) and TNFα (10 units/ml) were added and the cells were incubated at 37°C for 24 h. Then the culture medium was harvested and centrifuged. The supernatants were stored at −80°C for eicosanoid assay.

*Co-culture of macrophages and SMC:* Biosynthesis of $LTB_4$ during cell–cell interaction was studied by incubating fixed concentrations of $^3$H-AA labelled SMC with various numbers of alveolar macrophages. For co-culture experiments, $^3$H-labelled SMC ($5 \times 10^6$ cells) were further incubated with macrophages in the presence of IL-1β (5 units/ml) and TNFα (10 units/ml) at 37°C for 24 h following the ratios of SMC to macrophages: SMC alone, 100:1, 10:1, 1:1, 1:10, 1:100, macrophages alone, and macrophages alone but in the presence of IL-1β and TNF. After the incubation, the supernatants were collected, centrifuged and stored at −80°C until eicosanoid assay.

*Transformation of $LTA_4$ by macrophages:* Macrophages ($1 \times 10^6$ cells) in 35 mm wells were allowed to equilibrate for 5 min at 37°C in 1 ml HBSS/BSA and then incubated at 37°C for 15 min with $LTA_4$ at concentrations described in the figure legends. At the end of the incubation, 1 ml of ice-cold methanol was added and the medium was harvested, centrifuged and kept at −20°C for analysis by RIA for $LTB_4$.

*HPLC analysis:* The HPLC system utilized was from Waters associated (Milford, MA) using two pumps (6000A and 510) coupled to a Model 680 gradient controller. UV absorbance of the eluate was monitored using two serial detectors. Radioactivity was determined by mixing 1 ml of the effluent with 5–10 ml of Atomlight (NEN) and counting in a liquid scintillating counter.

The procedure used for the precolumn extraction/RP–HPLC of the supernatant was similar to that described previously,[19] with the six-port valve in the 'load' position. The sample, diluted to a total volume of 10 ml, was applied to a $C_{18}$ reverse-phase guard column (Nucleosil $C_{18}$, 10 μm, 10 mm, Macherey Nagal) located in the sample loop of the six-port switching valve (Rheodyne) which had been equilibrated with solvent A (2.5 mH $H_3PO_4$ in 15% methanol). A 3 μm filter was placed between the outlet of the pump and the six-port valve. The precolumn was then washed with 8 ml of solvent A. The AA metabolites, remaining on the precolumn, were injected by turning the six-port valve to the 'inject' position onto a Spherisorb ODS2 (5 μm, 4.6 × 150 mm) column (phase separation). RP–HPLC was carried out using a mobile phase consisting of a non-linear gradient starting with 30% solvent B (water/acetic acid 0.05, v/v, buffered to pH 5.7 with ammonium hydroxide) leading to 100% solvent C (65% acetonitrile–35% methanol) with the following program: 0–10 min, linear to 65%; 10–35 min, linear to 100% C; 35–55 min, linear to 30% C and isocratic until 70 min. The flow rate was 1 ml/min. Under these conditions, retention times (in min) of eicosanoids were as follows: 20-COOH-$LTB_4$, 5–6; 20-OH-$LTB_4$, 11.5; $LTC_4$, 18; $\Delta^6$-trans-$LTB_4$, 21.5; $LTB_4$, 22.5; 11-HETE, 23.5; 15-HETE, 24.5; 12-HETE, 38.5; 5-HETE, 40; arachidonic acid, 57.1.

*Radioimmunoassay of $LTB_4$:* The samples, acidified to pH 3.5, were extracted with Waters Sep-Pak $C_{18}$ (Milford, MA). Eicosanoids were eluted with methanol (5 ml). The solvent was pooled and evaporated to dryness under nitrogen. The residue was then dissolved in PBS for RIA assay. The detection limit of the radioimmunoassay for $LTB_4$ was 4 pg/ml.

### Results

*Induction of HETE synthesis in SMC by IL-1 and TNF:* Adherent rat vascular SMC, prelabelled with $^3$H-AA, were exposed to IL-1β (5 units/ml) or TNFα (10 units/ml) for 24 h. Following this incubation, the media were collected and then analysed by HPLC as described in Materials and Methods. As shown in Fig. 1, both IL-1β and TNFα induced a significant increase of lipoxygenase metabolites. These compounds eluted from an RP–HPLC column with retention times that corresponded to 15- and 5-HETE, and represented 10.9 ± 2.1% and 3.8 ± 0.9%, respectively, of the total radioactivity applied to the column. However, neither IL-1 nor TNF induced $LTB_4$ production. IL-1β or TNFα induced lipoxygenase metabolite formation in a dose-dependent manner (Fig. 1). The release of HETEs in response to IL-1β or TNFα, reached a plateau of release at concentrations above 100 units/ml. In comparison to the control, the amounts of HETEs in SMC treated with IL-1β or TNF were enhanced 6-fold and 4-fold, respectively. In all cases, the release of HETEs in response to IL-1β was significantly greater than that induced by TNFα (Fig. 2).

The response to IL-1 or TNF was also time-dependent having a lag phase of 8 h before significant generation of HETEs in both cases (Fig. 2).

The simultaneous addition of IL-1β and TNFα stimulated HETE release to a greater extent than did either agent alone (Fig. 2). The effect was additive and the sum of the HETEs released by both IL-1β and TNF always exceeded the amount of HETEs generated after separate addition of IL-1β or TNFα to SMC.

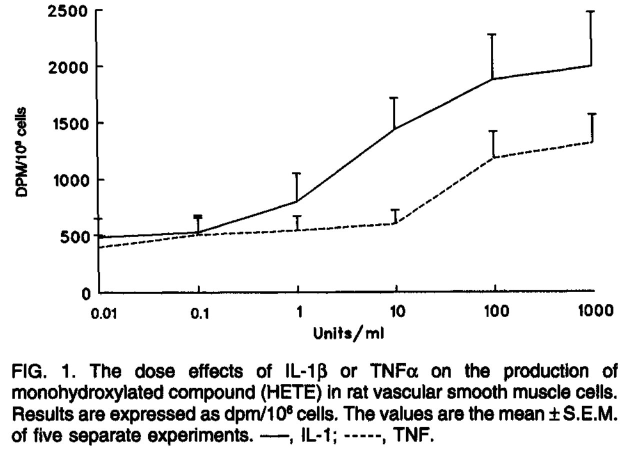

FIG. 1. The dose effects of IL-1β or TNFα on the production of monohydroxylated compound (HETE) in rat vascular smooth muscle cells. Results are expressed as dpm/$10^6$ cells. The values are the mean ± S.E.M. of five separate experiments. ——, IL-1; -----, TNF.

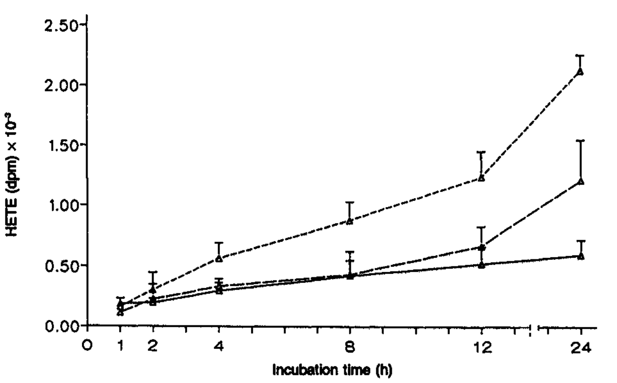

FIG. 2. The time course of monohydroxylated compound (HETE) production in rat vascular smooth vascular cells in response to IL-1β (5 units/ml), TNFα (10 units/ml), or IL-1β and TNFα. Results are expressed as dpm/$10^6$ cells and represent the mean ± S.E.M. of five separate experiments. ----, IL-1 + TNF; ---, IL-1 alone; —, TNF alone.

In addition, preincubation of SMC with NDGA ($10^{-5}$ M) inhibited recovery of HETEs induced by IL-1β and TNFα (90% and 95% inhibition for IL-1 and TNF, respectively). Metyrapone ($10^{-6}$ to $10^{-4}$ M), a cytochrome P450 inhibitor, did not modify the recovery of monohydroxylated compounds. The protein synthesis inhibitors cycloheximide ($10^{-5}$ M) and actinomycin ($10^{-5}$ M) also abolished HETEs production induced by IL-1 and TNF. In contrast, pretreatment of cells with aspirin ($10^{-6}$ to $10^{-5}$ M) inhibited the synthesis of cyclooxygenase metabolites (20 to 90%) and slightly increased HETEs recovery (Table 1).

In contrast to the augmentation of AA metabolites in SMC, incubation of macrophages with IL-1β (5 units/ml) or TNFα (5 units/ml) did not result in significant synthesis of $LTB_4$ (2.7 ± 0.5 pmol/$10^6$ cells) although the levels obtained from stimulated cells were higher than those obtained from unstimulated cells (2.5 ± 0.4 pmol/$10^6$ cells).

*Incubation of macrophages with $LTA_4$:* In order to synthesize $LTB_4$ from the small amounts of $LTA_4$ generated from adjacent cells, macrophages must be

**Table 1.** Effect of aspirin, NDGA, cycloheximide and actinomycin on the formation of monohydroxylated compounds in IL-1 and TNF stimulated rat vascular smooth muscle cells

| Sample | Monohydroxylated compounds (dpm/$10^6$ cells) | | |
|---|---|---|---|
| | IL-1 | TNF | IL-1 + TNF |
| Control | 1201 ± 215 | 570 ± 127 | 2370 ± 469 |
| Aspirin | 1375 ± 271 | 613 ± 136 | 2643 ± 437 |
| NDGA | 126 ± 21 | 103 ± 23 | 129 ± 47 |
| Metyrapone | 1172 ± 175 | 535 ± 139 | 2145 ± 513 |
| Cycloheximide | 235 ± 57 | 175 ± 27 | 376 ± 53 |
| Actinomycin | 269 ± 36 | 196 ± 39 | 257 ± 27 |

Adherent smooth muscle cells (5× $10^6$ cells) were incubated with IL-1β (5 units/ml) or/and TNF (10 units/ml) for 24 h after preincubation with aspirin ($10^{-5}$ M), NDGA ($10^{-5}$ M), cycloheximide ($10^{-5}$ M) and actinomycin ($10^{-5}$ M) for 1 h. The whole incubation mixture was extracted and analysed by HPLC as described in Materials and Methods. Results are expressed as mean ± S.E.M. from five experiments.

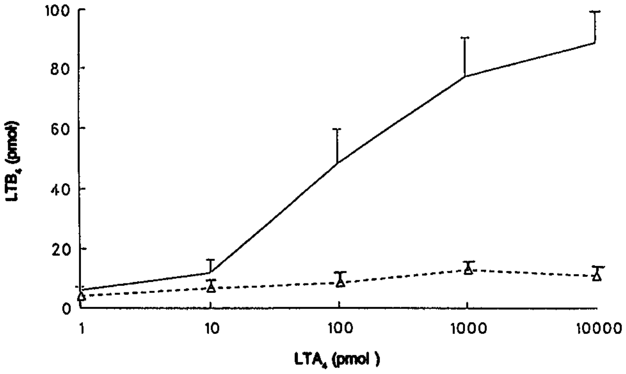

FIG. 3. Transformation of various concentrations of $LTA_4$ into $LTB_4$ by macrophages. Macrophages ($1\times10^6$ cells) in 35 mm wells with 1 ml of buffer were incubated at 37°C for 15 min in HBSS/BSA (4 g/l) with various concentrations of $LTA_4$ described in the legends. The samples were then analysed for $LTB_4$ by radioimmunoassay. Results represent the mean ± S.E.M. of six separate experiments. ——, AM present; ----, AM absent.

capable of converting exogenous supplies of $LTA_4$ into $LTB_4$. The ability of macrophages to utilize exogenous $LTA_4$ was examined by incubating macrophages ($1\times10^6$ cells) with exogenously added $LTA_4$. As shown in Fig. 3, macrophages were able to transform, in dose-dependent manner, extremely low concentrations of $LTA_4$ into $LTB_4$, but the amount of $LTB_4$ formed from the exogenous $LTA_4$ (1 to 10 000 pmol) in the absence of AM but in the presence of HBSS/BAS remained very low (5.6 to 10 pmol, as shown in Fig. 3). This finding suggested that transcellular synthesis of $LTB_4$ could occur when very limited amounts of $LTA_4$ were available to these cells.

*Transcellular $LTB_4$ synthesis in the co-culture of macrophages and SMC:* Biosynthesis of $LTB_4$ during cell–cell interaction was studied using co-incubation of SMC and macrophages in the presence of IL-1β or TNFα. As shown in Figs 4 and 5, incubation of SMC with various ratios of macrophages (100:1, 10:1, 1:1, 1:10, 1:00 and 1:1 000, SMC alone) resulted in significant amounts of $LTB_4$ in the supernatants. $LTB_4$ levels

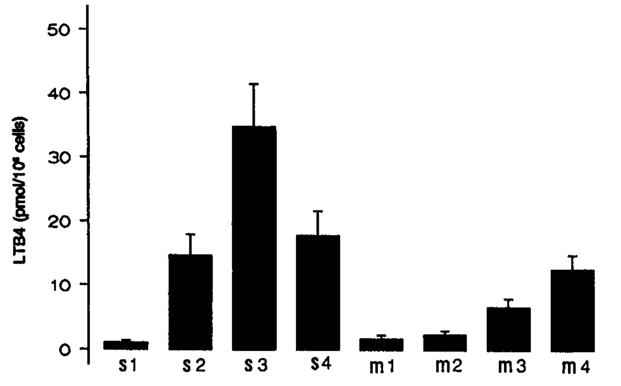

FIG. 4. The production of leukotriene $B_4$ in co-culture of macrophages and vascular smooth muscle cells (SMC) in the presence of IL-1β (5 units/ml) and TNFα (10 units/ml). S1, S2, S3 and S4 represent the various ratios of SMC to macrophages (SMC alone, 100:1, 10:1, 1:1 respectively). M1, M2, M3 and M4 represent the various ratios of macrophages to SMC (macrophages alone, and macrophages in the presence of IL-1β and TNF, 100:1, 10:1 respectively). The experimental procedure is described in Materials and Methods. Results are the mean ± S.E.M. of six separate experiments. $^*p < 0.05$, $^{**}p < 0.01$ (Student's paired *t*-test).

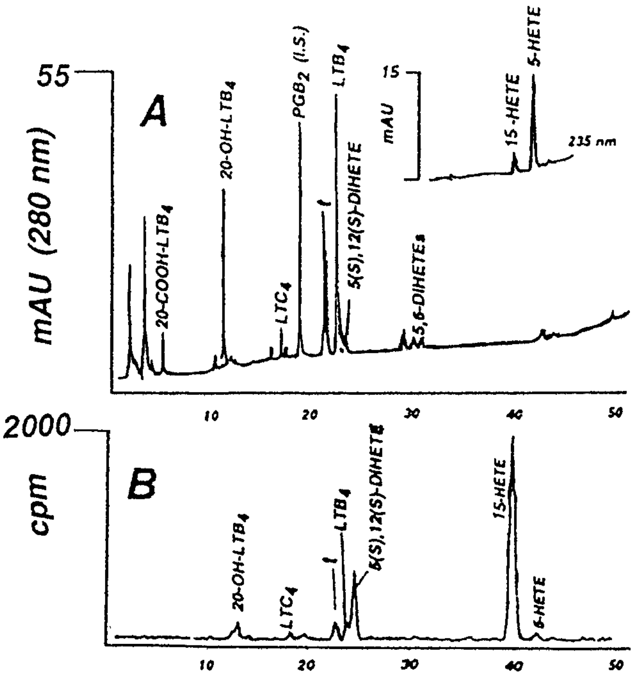

FIG. 5. (A) Reverse phase HPLC separation of lipoxygenase products in co-culture mixture as detected by UV absorption at 235 nm. (B) Radiochromatogram of metabolites derived from $^3$H-arachidonic acid generated in co-culture mixture as detected by HPLC. Rat vascular smooth muscle cells ($5\times10^6$) were incubated with alveolar macrophages ($5\times10^5$) in the presence of IL-1β (5 units/ml) and TNF (10 units/ml) for 24 h. The experimental procedures were described in Materials and Methods.

were 14.7 ± 2.5, 34.9 ± 6.7, 17.8 ± 4.3, 12.6 ± 2.9, 6.7 ± 2.7 and 2.4 ± 0.8 pmol/$10^6$ cells respectively. In addition, the preincubation of SMC with NDGA (10 μM), cycloheximide (10 μM), and actinomycin (10 μM) abolished $LTB_4$ production induced by IL-1 and TNF, when co-incubated with macrophages (Table 2). However, no $LTB_4$ was found when IL-1- and TNF-treated SMC were co-incubated with NDGA pretreated macrophages.

### Discussion

In this study, the transcellular synthesis of $LTB_4$ during cell–cell interaction between IL-1β and TNFα activated vascular SMC and macrophages has been

**Table 2.** Effect of aspirin, NDGA, cycloheximide and actinomycin on $LTB_4$ formation in co-culture of macrophages with IL-1 and TNF stimulated rat vascular muscle smooth cells

| Sample | $LTB_4$ (pmol/$10^6$ cells) |
|---|---|
| IL-1 | 34.9 ± 6.7 |
| Aspirin + IL-1 | 37.2 ± 5.9 |
| NDGA + IL-1 | 4.7 ± 1.7 |
| Cycloheximide + IL-1 | 4.9 ± 2.5 |
| Actinomycin + IL-1 | 4.7 ± 2.1 |

Adherent smooth muscle cells ($5\times10^6$ cells) were incubated with $5\times10^5$ macrophages in the presence of IL-1β (5 units/ml) and TNF (10 units/ml) for 24 h after preincubation with aspirin ($10^{-5}$ M), NDGA ($10^{-5}$ M), cycloheximide ($10^{-5}$ M) and actinomycin ($10^{-5}$ M) for 1 h. The whole incubation mixture was extracted and analysed by HPLC as described in Materials and Methods. Results are expressed as mean ± S.E.M. from five experiments demonstrated for the first time. The incubation of individual SMC with IL-1β or TNFα did not produce $LTB_4$. Neither IL-1β or TNFα induced significant $LTB_4$ production in macrophages incubated at 37°C for 24 h. However, using co-culture of SMC with various ratios of macrophages in the presence of IL-1β and TNFα, it was found that levels of $LTB_4$ were greatly increased compared with macrophages alone.

In accordance with previous reports on rabbit chrondrocytes,[20] rheumatoid synovial cells,[21] and human glomerular mesengial cells,[22] both IL-1β and TNFα induced a dose- and time-dependent formation of eicosanoids. The amount of HETEs was significantly greater than the additive effects of the two cytokines alone. The mechanism by which IL-1β and TNFα activated the HETEs synthesis is not known. Many reports have indicated that IL-1β and TNFα cause an up-regulation of phospholipase $A_2$ and cyclooxygenase-2, which, unlike cyclooxygenase-1, metabolize arachidonic acid not only to prostaglandins but also to a significant extent to 11-, 15- and 5-HETE.[20,23,24] The increase of HETE synthesis in SMC cells might also be due to the increase of enzyme protein synthesis, such as cyclooxygenase and/or phospholipase $A_2$ in IL-1β or TNFα stimulated cells because cycloheximide, a protein inhibitor, inhibited HETE increase induced by IL-1β and TNFα in SMC, but the mechanism remains to be elucidated.

The synthesis of $LTB_4$ involves a complex series of reactions within macrophages. A limiting factor for $LTB_4$ biosynthesis was found to be the availability of the unstable intermediate, $LTA_4$.[6] However, the generation of $LTA_4$ depends upon activation of 5-lipoxygenase as well as the presence of its substrate, arachidonic acid.[25] The production of LTs by human polymorphonuclear leukocytes has been known to be significantly influenced by adjacent cells possessing distinctly different metabolic properties.[2–5] In this study, macrophages transformed very low concentrations of exogenous $LTA_4$ into $LTB_4$ suggesting that macrophages could metabolize $LTA_4$ into biologically active $LTB_4$. The co-culture of SMC with various ratios of macrophages greatly increased $LTB_4$ synthesis when compared with very low levels of $LTB_4$ produced by individual cells. The highest augmentation in $LTB_4$ was obtained when the ratio of SMC to macrophages was 10:1. Because IL-1β and TNFα did not induce a significant $LTB_4$ production either in SMC alone or in macrophages alone, and because the pretreatment of SMC cells and macrophages with the lipoxygenase inhibitor NDGA and protein synthesis inhibitors, blocked $LTB_4$ formation, it could be concluded that cell–cell interaction could be responsible for this augmentation, i.e. macrophages utilizing SMC-derived $LTA_4$ or its precursor, 5-HPETE for its syn-thesis of $LTB_4$ during co-incubation of SMC and macrophages. According to the cell cooperation classification of Marcus, $LTB_4$ biosynthesis could come from the interaction of type IA (cells can share a common precursor synthesized by different cells).[24]

However, the mechanism of $LTB_4$ transcellular synthesis is certainly more complex. Irvine[25] has demonstrated that arachidonic acid availability is a major limiting factor for LTs synthesis. Furthermore, several studies have suggested that a transfer of arachidonic acid takes place in *in vitro* cell–cell cooperation such as during platelet–neutrophil interaction.[8,10,25,26] For example, Palmentier and Borgeat[7] have presented evidence that thrombin activated platelets offer free arachidonic acid to neutrophils that are utilized for $LTB_4$ synthesis. Antoine *et al.*[8] have also found that neutrophils can utilize platelet-derived arachidonic acid for the formation of the 5-lipoxygenase product.[8] Therefore, the possibility that other mechanisms are responsible for $LTB_4$ synthesis cannot be excluded from the present data.

The transcellular synthesis of $LTB_4$ between vascular SMC and macrophages may have an important physiological and pathophysiological significance. The lipoxygenase products of AA metabolism are involved in various aspects of inflammation and atherosclerotic lesions.[27] For example, the intimal accumulation of smooth muscle cells in rabbit carotid arteries, an early stage of atherosclerosis, is inhibited by dexamethasone, which prevents formation of both cyclooxygenase and lipoxygenase metabolites, but is not inhibited by nonsteroidal anti-inflammatory drugs such as indomethacin.[28,29] The transcellular $LTB_4$ synthesis could be involved in the genesis of atherosclerosis and other vascular diseases because of its action of initiating vascular inflammation, promoting vascular constriction and proliferation as well as platelet aggregation.

### References

1. Samuelsson B, Serhan CN. On the formation and biological role of leukotrienes and lipoxins. In: Garaci E, Paoletti R, Santorro MG, eds. *Prostaglandins in Cancer Research*, Berlin Heidelberg: Springer, 1987: 3–11.
2. McGee GE, Fitzpatrick FA. Erythrocyte-neutrophil interaction: formation of leukotriene $B_4$ by transcellular biosynthesis. *Proc Natl Acad Sci* 1986; **83**: 1349–1353.
3. Claesson HE, Haëggstrom J. Human endothelial cells stimulate leukotriene synthesis and convert granulocyte released leukotriene $A_4$ into leukotriene $B_4$, $C_4$, $D_4$ and E4. *Eur J Biochem* 1988; **173**: 93–100.
4. Feinmark SJ, Cannon PJ. Endothelial cells leukotriene $C_4$ synthesized by polymorphonuclear leukocytes. *J Biol Chem* 1986; **261**: 16466–16472.
5. Maclouf J, Murphy RC. Transcellular metabolism of neutrophil derived leukotriene $A_4$ by human platelets. A potential source of leukotriene $C_4$. *J Biol Chem* 1988; **263**: 174–181.
6. Maclouf J, Lurohy RC. Transcellular sulfidopeptide leukotriene biosynthetic capacity of vascular cells. *Blood* 1989; **74**: 703–707.
7. Palmantier R, Borgeat P. Thrombin-activated platelets promote leukotriene $B_4$ synthesis in polymorphonuclear leukocytes stimulated by physiological agents. *Br J Pharmacol* 1991; **103**: 1909–1916.
8. Antoine C, Murphy RC, Henson PM, Maclouf J. Time-dependent utilization of platelet arachidonic acid by the neutrophil in formation of 5-lipoxygenase in platelet-neutrophil co-incubations. *Biochim Biophys Acta* 1992; **1128**: 139–146.
9. Grimminger F, Kreusler B, Schneider V, Becker G, Seeger W. Influence of microvascular adherence on neutrophil leukotriene generation: evidence for the cooperative eicosanoid synthesis. *J Immunol* 1990; **144**: 1866–1872.
10. Marcus AJ, Broekman MJ, Safier LB, *et al.* Formation of leukotrienes and other hydroxy-acids during platelet-neutrophil interaction *in vitro*. *Biochem Biophys Res Commun* 1982; **109**: 130–145.

11. Johnston RB Jr. Monocytes and macrophages. *N Engl J Med* 1988; **318**: 747–775.
12. Bigby TD, Holtzman MT. Enhanced 5-lipoxygenase activity in lung macrophages compared to monocytes from normal subjects. *J Immunol* 1987; **138**: 1546–1550.
13. Balter MS, Toews GB, Peters-Golden M. Different patterns of arachidonate metabolism in autologous human blood monocytes and alveolar macrophages. *J Immunol* 1989; **142**: 602–608.
14. Elias JA, Ferro TJ, Rossman MD, *et al.* Differential prostaglandin production by unfractionated and density fractionated human monocytes and alveolar macrophages. *J Leukocyte Biol* 1987; **42**: 114–121.
15. Bonney RJ, Humes JL. Physiological and pharmacological regulation of prostaglandin and leukotriene production by macrophages. *J Leukocyte Biol* 1984; **35**: 1–10.
16. Nigon F, Rouis M, Forest SJ, Chapman MJ. Native low-density lipoproteins stimulate leukotriene $B_4$ production by human monocyte-derived macrophages. *Biochim Biophys Acta* 1991; **1083**: 230–234.
17. Maycock AL, Anderson MS, DeSousa DM, Kuehl FA. Leukotriene $A_4$: preparation and enzymatic conversion in a cell-free system to leukotriene $B_4$. *J Biol Chem* 1982; **257**: 13911–13922.
18. Peters-Golden MJ, Bathon J, Flores R, Hirata F, Newcombe DS. Glucocorticoid inhibition of zymosan-induced arachidonic acid release by rat alveolar macrophages. *Am Rev Respir Dis* 1984; **130**: 803–809.
19. Powell WS. Precolumn extraction and reversed-phase high-pressure liquid chromatography of prostaglandins and leukotrienes. *Anal Biochem* 1987; **164**: 117–131.
20. Kerr SJ, Stevens RM, Davis GL, McLaughlin AL, Harris RR. Effects of recombinant IL-1β on phospholipase $A_2$ activity, phospholipase 42 mRNA levels, and eicosanoid formation in rabbit chrondrocytes. *Biochem Biophys Res Commun* 1989; **165**: 1079–1084.
21. Dinarello CA. Biology of interleukin-1. *FASEB J* 1988; **2**: 108–115.
22. Topley N, Floege J, Wessel K, *et al.* Prostaglandin E2 production is synergistically increased in cultured human glomerular mesangial cells by combinations of IL-1 and tumour necrosis factor-α. *J Immunol* 1990; **143**: 1989–1995.
23. Bomalaski JS, Steiner MR, Simon PL, Clark MA. IL-1 increases phospholipase $A_2$ activity, expression of phospholipase $A_2$-activating protein, and release of einoleic acid from the murine Y helper cell line EL-4. *J Immunol* 1992; **148**: 155–160.
24. Raz A, Wyche A, Siegel N, Needeman P. Regulation of fibroblast cyclooxygenase synthesis by interleukin-1. *J Biol Chem* 1988; **263**: 3022–3028.
25. Irvine RF. How is the level of free arachidonic acid controlled in mammalian cells? *Biochem J* 1982; **204**: 3–16.
26. Marcus AJ. Transcellular metabolism of eicosanoids. *Prog Hemost Thromb* 1986; **8**: 127–142.
27. Davies P, Bailey PJ, Goldenberg MM, Ford-Hutchinson AW. The role of arachidonic acid oxygenation products in pain and inflammation. *Ann Rev Immunol* 1984; **2**: 335–357.
28. Hirosumi J, Nomoto A, Ohkubo Y *et al.* Inflammatory responses in cuff-induced atherosclerosis in rabbits. *Atherosclerosis* 1987; **64**: 243–254.
29. Bailey JM, Butler J. Anti-inflammatory drugs in experimental atherosclerosis. I Relative potencies for inhibiting plaque formation. *Atherosclerosis* 1973: **17**: 515–522.

ACKNOWLEDGEMENTS. This work was supported by grants from EPR Aquitaine and Squibb Laboratoire. We wish to thank Drs Jacky Larrue and Monique Parant for critically reviewing our manuscript.

**Received 2 February 1994; accepted in revised form 14 April 1994**
